# Periodontal evaluation of palatally impacted maxillary canines treated by closed approach with ultrasonic surgery and orthodontic treatment: a retrospective pilot study

**DOI:** 10.1038/s41598-021-82510-y

**Published:** 2021-02-02

**Authors:** Camilla Grenga, Rosanna Guarnieri, Vittorio Grenga, Mauro Bovi, Serena Bertoldo, Gabriella Galluccio, Roberto Di Giorgio, Ersilia Barbato

**Affiliations:** 1 Rome, Italy; 2 Department of Oral and Maxillofacial Sciences, School of Dentistry, Sapienza, University of Rome, Rome, Italy

**Keywords:** Oral diseases, Biomedical engineering

## Abstract

Aim of this study is the evaluation of the periodontal status of impacted canines treated by closed approach with ultrasonic surgery and orthodontic treatment compared with contralateral spontaneously erupted teeth. The periodontal conditions of the teeth adjacent to the canines (lateral incisors and first premolar) were also considered. 17 patients (9 females and 8 males; mean age: 15.2 years) with unilateral palatal impaction of maxillary canine were selected. All patients were treated by closed-flap surgery with ultrasonic instruments. Periodontal status was evaluated by assessing probing depth (PD), gingival recession and width of keratinized tissue (KT) 4.6 months after the end of the orthodontic treatment, on average. Test group was composed by impacted elements and adjacent teeth and control group by contralateral spontaneously erupted canines and adjacent teeth. Student's t-test was used to compare test and control group values of PD and width of KT. Significance threshold for Student's t-test was set at *p* < 0.05. The average probing depth values show no significant clinical differences between the test and control groups. Probing depths recorded at the mesiovestibular and distopalatal sides of the impacted canine were statistically significant compared to the control elements (*p* < 0.05). No gingival recession was detected on the treated canines. The measurement of KT did not differ significantly between the test and the control groups. In conclusion, the ultrasonic surgery for disinclusion associated with a closed approach and orthodontic traction allows the alignment of an impacted palatal canine without damaging the periodontium.

## Introduction

The upper permanent canine is the tooth with the second highest incidence of impaction after the third molar, with a prevalence between 1 and 3%. The inclusion of the upper canine shows a predilection for the palatal side and has a prevalence almost three times greater in female patients^1,2^.

The management of the impacted canines includes early interceptive measures or late interventions such as extraction, transplantation and surgical exposure of the impacted canine crown with subsequent orthodontic alignment of the tooth^3^.

The failure to reposition this element can compromise the aesthetic and functional aspect of the patient. It could also entail root resorption of the adjacent permanent teeth and lead to a follicular cyst^4^.

The surgical orthodontic treatment of palatally impacted canines can be carried out by means of open-flap or closed-flap surgery followed by orthodontic traction of the impacted canine. The available scientific evidence suggests that there is no difference in surgical and periodontal outcomes between the two methods^5,6^. In recent times, ultrasonic surgery has been introduced as a new piezoelectric technology in the field of dental bone surgery. Piezosurgery is a relatively new technique utilizing the microvibrations of scalpels at ultrasonic frequency to perform safe and effective osteotomies^7^. When used properly, piezosurgery causes less damage at the structural and cellular level compared to other techniques^8^. Moreover, the formation of the new bone is more rapid after piezosurgery than after surgery performed with the rotating drill^9^. This is possible thanks to one of the foundations of this surgery: the selective cutting towards hard tissues with respect to soft tissues. Through selective cutting it is also possible to distinguish between bone, root cement and enamel^10^. This allows not to damage the adjacent dental structures and in particular the cemento-enamel junction (CEJ) of the tooth. The preservation of the CEJ appears to be indispensable for a correct conservation of the periodontal structures, as well as to avoid the ankylosis and to facilitate a physiological movement of the tooth^11,12^.

The aim of this work was to evaluate the periodontal status of impacted canines treated by closed approach with ultrasonic surgery and orthodontic treatment compared with contralateral spontaneously erupted teeth. The periodontal conditions of the teeth adjacent to the canines (lateral incisors and first premolar) were also considered.

## Materials and methods

### Sample

The study was carried out as a retrospective study. The investigation was reviewed and approved by the regional Ethical Review Board of the Umberto I General Hospital (No. 3755).

The patients were screened and treated in the Department of Oral and Maxillofacial Sciences between 2013 and 2019 according to a standardized and existing protocol that provided for:

- an initial visit with the orthopantomography,
- a closed-flap surgery with ultrasonic instruments followed by orthodontic traction of the impacted canine,
- a follow-up visit after 4–6 months after the end of their orthodontic treatment.

In sample size calculation we performed Lehr’s formula for a power of 90% and a two-sided significance level of 0.05; the minimum number of participants necessary for the validity of the study was 16.

From the original study sample of 101 subjects with palatal impaction of maxillary canine, a group of 17 patients (9 females and 8 males, ages ranged from 11.11 to 23.4 years, mean: 15.1 years) has been selected. The inclusion criteria were as follows: (1) age up to 25 years, (2) good level of oral hygiene assessed by plaque index (PI) according to Silness and Löe^13^. The exclusion criteria were as follows: (1) bilateral palatally canine impaction, (2) previous orthodontic treatment, (3) metabolic disorders or other conditions capable of influencing the treatment (Table 1).

**Table 1:** Description of the sample group.

	Mean	SD
Age at the time of surgery (years)	15.1	3.1
Treatment time from surgery to debonding (months)	12.4	3.0
Recall for periodontal evaluation (months)	4.6	0.8

*SD* standard deviation.

All selected patients were treated with ultrasonic surgery and closed approach. The contralateral canine, which had erupted spontaneously, served as a control during the study.

All patients were treated by the same operators (surgeon and orthodontist).

### Radiographic parameters

**Figure 1:**
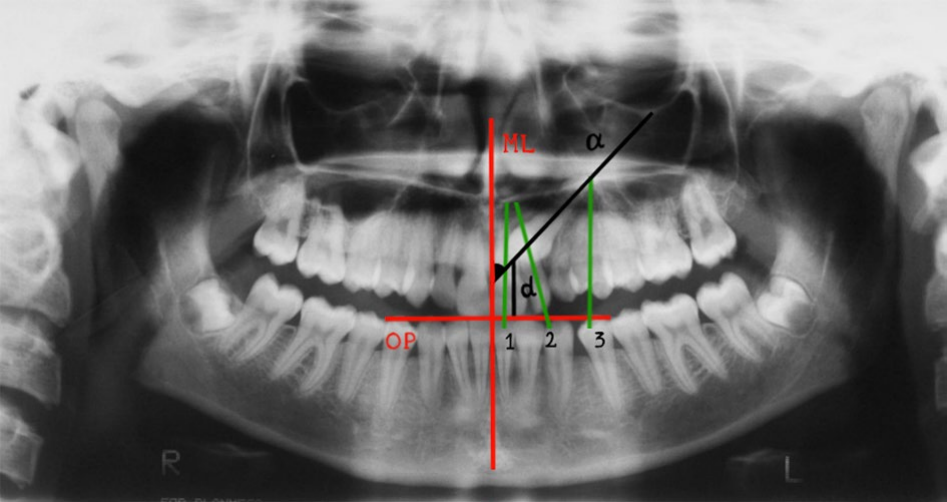
Schematic illustration showing the criteria used to define the position of the impacted maxillary canine: *Angle α* = the angle formed between the long axis of the impacted canine and the inter-incisor median line; *Distance* *d* = the distance between the peak of the impacted cuspid and the occlusal plane; *Sectors* = the area in which the impacted cuspid is located, divided in *sector 1* between the inter-incisor median line and the long axis of the central incisor, *sector 2* between the long axes of the lateral and central incisors, *sector 3* between the long axes of the lateral incisor and the first premolar.

The position of the impacted canines was evaluated on an orthopantomography using the criteria proposed by Ericson and Kurol (1988)^14^ in the simplified version of Baccetti et al. (3 Sectors instead of 5)^15^:

- Angle α = normal value = 20–53 degrees
- Distance *d* = normal value = 7–26 mm
- Sectors = 1, 2, 3 (Fig. 1).

### Surgical-orthodontic treatment

All the patients were submitted to the same surgical-orthodontic treatment. The initial orthodontic treatment consisted of the application of stabilization devices (transpalatal arch, rapid palatal expander) on the upper arch in order to minimize the side effects generated by the traction of the impacted canine and to create space for the subsequent repositioning of the canine.

**Figure 2:**
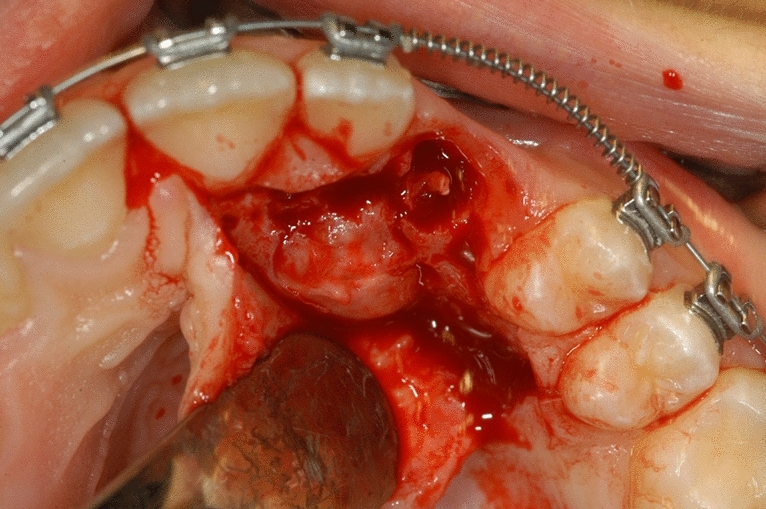
Elevation of a mucoperiosteal full-thickness palatal flap.

**Figure 3:**
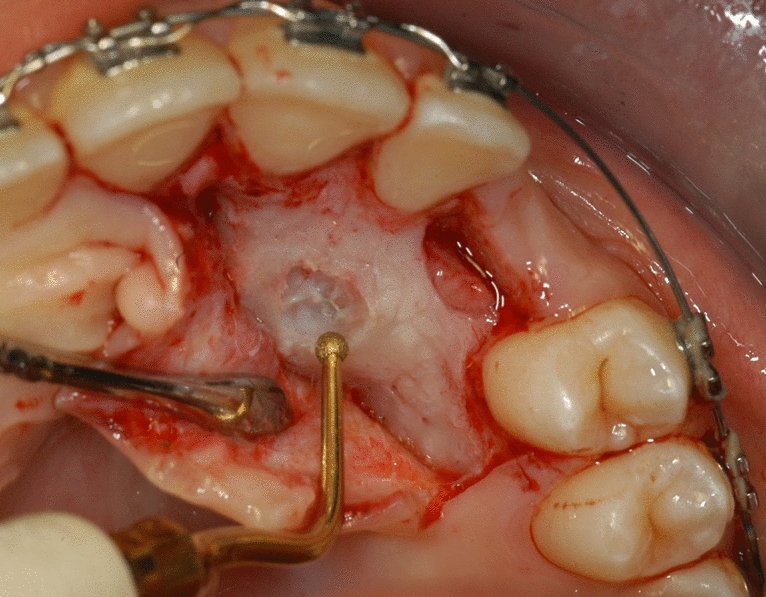
The cortical bone overlying the canine is removed using an ultrasonic device with a diamond-coated ball insert.

**Figure 4:**
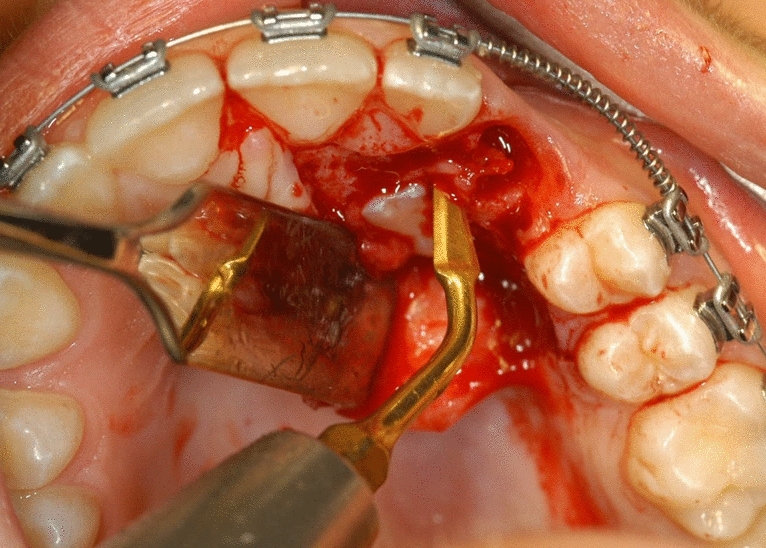
The pericoronal bag is removed using an angled or straight lanceolate insert.

**Figure 5:**
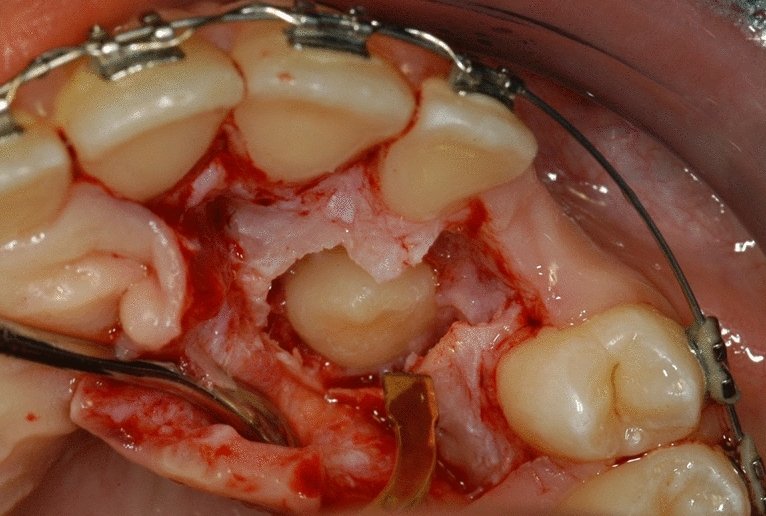
A guide tunnel is created using the saw insert.

**Figure 6:**
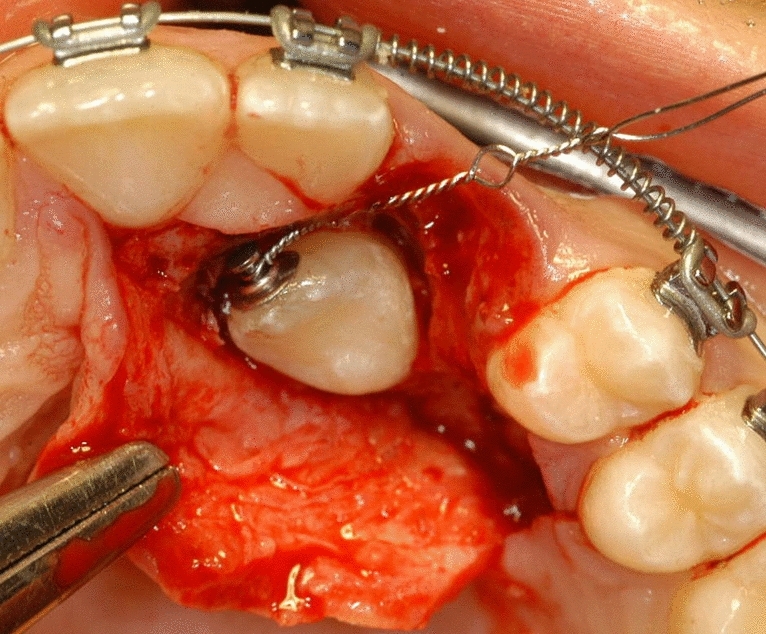
On the vestibular surface of the canine a traditional orthodontic button connected to the arch is applied using a metal ligature.

**Figure 7:**
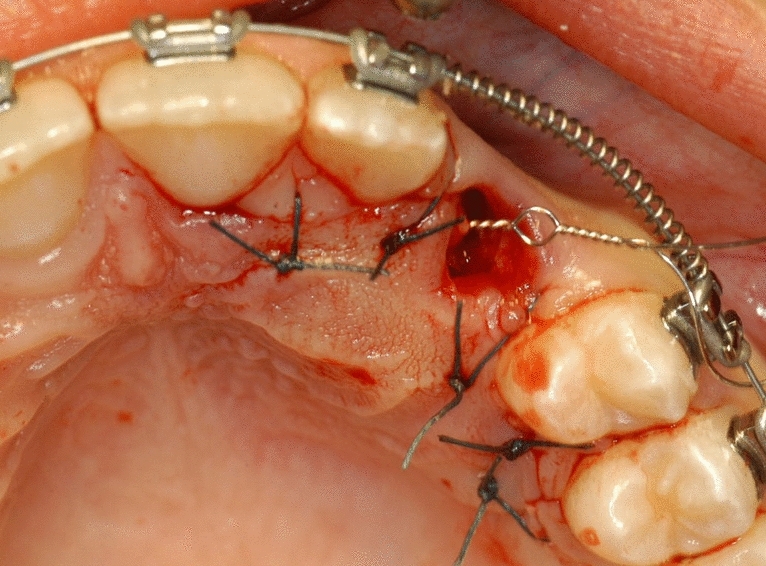
The flap sutured. Orthodontic traction starts immediately after surgery by progressive activation of metal ligatures connected to a Ni–Ti .018 arch.

Surgical treatment resulted in the elevation of a mucoperiosteal full-thickness palatal flap (Fig. 2). Once the flap was elevated, the cortical bone overlying the canines was removed using an ultrasonic device with a diamond-coated ball insert (Fig. 3). The pericoronal bag was removed using an angled or straight lanceolate insert (Fig. 4) and a guide tunnel was created using the saw insert (Fig. 5). On the vestibular surface of the canines a traditional orthodontic button connected to the arch was applied using a metal ligature (Fig. 6). The flap was then sutured (Fig. 7).

Orthodontic traction started immediately after surgery by progressive activation of metal ligatures connected to a Ni–Ti 0.018 arch. Traction has been reactivated every month. The average time of canine repositioning was 6 ± 2 months.

### Periodontal evaluation

Patients were recalled for follow-up 4.6 months after the end of their treatment, on average. The post-treatment periodontal examination was carried out with a PCP 15 periodontal probe and each measurement was taken three times in order to avoid method error.

The periodontal status of the first premolar, the canine, and the lateral incisor was evaluated by assessing probing depth (PD), gingival recession (GR), and width of keratinized tissue (KT) on the impacted and contralateral canine quadrant. The test group was composed by impacted canines and the adjacent teeth, while the control group was composed by the contralateral spontaneously erupted upper canines and adjacent teeth.

PD was measured from the gingival margin to the base of the pocket, at six tooth surface points: mesiovestibular (MV), midvestibular (V), distovestibular (DV), mesiopalatal (MP), midpalatal (P), and distopalatal (DP).

The same technique was also used to evaluate gingival recession, measuring the distance between the CEJ and the gingival margin.

The width of KT was measured as the distance between the gingival margin and the mucogingival junction on the three points of the vestibular side of the canine crown (MV, V, DV), the MV point of the first premolar and the DV of the lateral incisor.

## Statistical analysis

A Fisher test was carried out to evaluate the homogeneity between the variances of the groups to be compared, which in this case confirms the homoscedasticity.

Subsequently, for each tooth and for each site, a comparison was made between the quantitative distributions of the test group samples with control group samples through a paired Student t-test with the hypothesis of homoscedasticity.

Student's t-test was used to compare test and control group values of PD, GR and width of KT. The significance threshold for Student's t-test was set at *p* < 0.05. It has been used the paired Student’s t-test.

The Pearson index was used to evaluate the correlation between PD and the other factors such as gender, age and pretreatment radiographic variables (alpha angle, distance D, sector S).

## Results

The pre-treatment radiographic parameters collected from the sample are summarized in Table 2. The distribution of the position of the impacted canines is 59% in sector 1, 41% in sector 2 and 0% in sector 3.

**Table 2:** Pretreatment radiographic parameters.

	Mean	SD	Minimum–Maximum
Angle α (°)	38.6	18.9	20–80
Distance *d* (mm)	18.3	3.2	14–25
	Sector 1	Sector 2	Sector 3
Frequency	10	7	0

*SD* standard deviation.

The average probing depth values calculated to the second decimal (Table 3) show no significant clinical differences between the test and the control groups. Probing depths recorded at the MV and DP aspects of the impacted canine (*p* < 0.05) are statistically significant compared to the control elements.

**Table 3:** T-test and *p* value of the PD.

	Test	Group	Control	Group		*p* value
	Mean	SD	Mean	SD	Diff	
**Canine**						
MV	2.20	0.54	1.79	0.52	0.42	0.02
V	1.73	0.71	1.64	0.79	0.09	0.79
DV	1.97	0.47	2.21	0.41	− 0.24	0.22
DP	2.26	0.62	1.79	0.56	0.48	0.02
P	1.73	0.52	1.89	0.78	− 0.16	0.80
MP	1.85	0.48	1.96	0.74	− 0.11	0.58
**Lateral incisor**						
MV	2.09	0.75	2.50	0.68	− 0.41	0.08
V	1.71	0.73	1.62	0.47	0.08	0.63
DV	2.00	0.64	1.73	0.65	0.27	0.38
DP	1.85	0.48	1.76	0.55	0.09	0.76
P	1.71	0.52	1.79	0.62	− 0.08	0.59
MP	1.91	0.49	1.87	0.51	0.04	0.67
**First premolar**						
MV	2.23	0.73	2.18	0.67	0.06	0.72
V	1.53	0.74	2.05	0.78	− 0.52	0.06
DV	1.88	0.85	2.04	0.58	− 0.15	0.38
DP	1.85	0.68	1.96	0.55	− 0.11	0.72
P	1.76	0.71	1.86	0.44	− 0.09	1.00
MP	1.79	0.62	1.93	0.53	− 0.13	0.23

*SD* standard deviation.

Gingival recessions were detected on 2 normally erupted canines, on 3 premolars close to the treated canines and on 2 lateral incisors (1 near the treated canine and 1 near the normally erupted canine). The most pronounced recession (4 mm) was found on a normally erupted canine.

Differences in gingival recession between test and control group were non-significant (Table 4).

**Table 4:** T-test and *p *value of the GR.

	Test	Group	Control	Group		*p* value
	Mean	SD	Mean	SD	Diff	
**Canine**						
MV	0	0	0	0	0	–
V	0	0	0.29	0.96	-0.29	0.23
DV	0	0	0	0	0	–
DP	0	0	0	0	0	–
P	0	0	0	0	0	–
MP	0	0	0	0	0	–
**Lateral incisor**						
MV	0	0	0	0	0	–
V	0	0	0	0	0	–
DV	0.06	0.24	0.06	0.24	0	1
DP	0	0	0	0	0	–
P	0	0	0	0	0	–
MP	0	0	0	0	0	–
**First premolar**						
MV	0.41	1.14	0	0	0.41	0.16
V	0.06	0.24	0	0	0.06	0.32
DV	0	0	0	0	0	–
DP	0	0	0	0	0	–
P	0	0	0	0	0	–
MP	0	0	0	0	0	–

*SD* standard deviation.

The width of KT does not differ significantly between the test and the control groups (Table 5).

**Table 5:** T-test and p-value of the KT.

	Test	Group	Control	Group		*p* value
	Media	DS	Media	SD	Diff	
**Canine**						
MV	5.12	0.99	5.35	0.86	− 0.23	0.26
V	4.82	1.09	4.53	1.47	0.29	0.38
DV	5.21	1.10	5.18	0.75	0.03	0.91
DV	4.97	1.42	4.82	0.1	0.15	0.69
MV	4.59	1.63	4.85	1.13	− 0.26	0.50

*SD* standard deviation.

The correlation study, carried out through the Pearson correlation index, confirms that there is no association between PD and other factors, such as gender, age of the patient at the start of treatment and the initial position of the canines evaluated through radiography.

## Discussion

The treatment of impacted canine requires a multidisciplinary approach in which surgery is a fundamental step. The aim is the alignment of the tooth without periodontal damage^16^.

Previous studies on this topic used a traditional surgical technique for disinclusion. Only one study focused on the results and effects of piezosurgery on soft tissue^17^.

Our results do not differ from previous studies on the traditional technique. In the test group, the average probing depth in the mesiovestibular and distopalatal sites of the impacted canine showed a statistically significant increase compared to the values recorded in all other sites of the same tooth, and also compared to the average of the same sites in the control group.

Hansson observed increased depth of the mesiopalatal and mesiovestibular pockets of corrected canines^18^. Wisth detected that the pocket on the distal surface was significantly deeper in the experimental group^19^. The Authors hypothesized that the severe periodontal change may be due to different factors such as less favourable hygienic conditions on the experimental teeth, the formation of a pressure zone during the uprighting, or the radical surgical exposure of the crown. Another study by Woloshyn reported that PD was deeper at the mesial and distal sides of the impacted canine and that this could be attributable to a more aggressive surgical exposure^20^. The work of Hansson showed greater mesial probing depth of the canines on the treated side. These findings might be explained by the fact that the palatally impacted canine had not been completely uprighted during orthodontic treatment and it had reached occlusion with its attachment at more apical level^21^. On the other hand, Wisth noted deeper distal pockets on the treated canine (*p* < 0.05), perhaps as a result of orthodontic pressure forces^19^. Also Szarmach reported increased pocket depths at the mesial and distal sides of previously palatally impacted canines^22^. Zasciuriskiene found that surgical-orthodontic treatment affected pocket depth at the MP site on the impacted canine, which was greater than on control canines^23^.

It is important to underline that, in our study, the increase in probing depth in the mesial and distal sites of the previously impacted teeth was of minor relevance from the clinical point of view: the average PD did not exceed 3 mm and in one individual case PD was 4 mm. Considering the mean age of the patients, these results could improve during the 3 year follow up, as evidenced by Crescini^24^. This pattern of improvement over the 3 years, without attachment loss, is due to the apical migration of gingival margin and reduction of the free gingiva. This is also shown by the finding of increased probing depths at the mesial and distal sites and reduced probing depths at the medial sites of the test group’s teeth. This pattern probably indicates a process of remodelling and regeneration of the interdental papilla.

Regarding the average probing depth of the lateral incisors adjacent to the treated canine, we found slightly greater probing depths in the distal sites. These results are in agreement with those of Woloshyn, Hansson and Szarmach, which may be explained by reduced cleanliness^20–22^. According to Woloshyn, appliances and attachments frequently cross the embrasure distal to the lateral incisors during distal movement and rotation of the canines, possibly increasing the tendency to inflammation^20^. Szarmach explained that the process of alignment is accompanied by changes in the structure of periodontal tissue^22^.

Concerning the adjacent premolars, we found neither statistically nor clinically significant changes in PD among the test group compared to the control group. In a study by Caprioglio, instead, PD recorded at the vestibular surface of the lateral incisor (*p* < 0.05) and at the mesiopalatal/mesiolingual site of the first premolar were statistically significant in comparison with the control elements^25^.

These findings support those of other research studies. Zasciuriskiene found pocket depth differences between teeth adjacent to the impacted canine and suggest that these results mainly depend on the surgical-orthodontic treatment, which exposes the adjacent teeth to larger intrusive forces and root torque during extrusion, distal movement and alignment of the impacted canine^23^.

The study of correlations, carried out through the Pearson correlation index, confirmed that there is no association between PD and other factors, such as sex, age of the patient at the beginning of treatment and the initial position of the affected canines detected by radiographic evaluation through criteria proposed by Ericson and Kurol. This result is in line with those of Crescini, Nieri and Smailiene: the final periodontal status did not depend on age, sex or pre-treatment radiographic features^26–28^.

Considering the overlap of the periodontal results obtained in our study with those found in the literature on traditional surgery, the innovative findings concern the advantages of the piezoelectric surgical technique.

Piezoelectric surgery is a less aggressive surgical procedure than the traditional one and it responds to the need for higher levels of precision and safety in bone surgery^12^. The preservation of the bone structure, observed after the use of the ultrasonic technique, seems to improve cellular reactivity thus favouring the healing process of the traumatized mineralized tissues^9,29,30^. In some studies concerning the extraction of impacted third molars, piezosurgery caused less injury of bone tissues compared to conventional rotary instruments thanks also to reduction of overheating and bleeding resulting from the generation of a cavitation effect^31–33^. Piezosurgery insured a better blood supply resulting in lower incidence of postoperative inflammation and minor postoperative complications such as pain, trismus, swelling^34^.

Furthermore, a precise surgical cut is extremely important when the crown of a palatally impacted maxillary canine is close to the roots or crowns of the central and lateral incisors, as it is often the case. Traditional burs do not distinguish between the mineralization and hardness of the bone, the radicular cementum, and the enamel. Piezoelectric surgery is precise enough to account for such differences, thus avoiding damage to the adjacent teeth^35^.

## Conclusion

1. The use of ultrasonic surgery for disinclusion associated with a closed approach and orthodontic traction allows the alignment of an impacted palatal canine without damaging the periodontium.
2. Piezosurgery can be considered an alternative to traditional surgery.

## Limitations of the study

The study presents several limitations such as the small study population and the retrospective design; retrospective studies do not have the power as the prospective randomized ones to control bias.

Due to the advantages of Piezosurgery and considering both the limitations discussed previously and the lack of similar studies in literature, it would be interesting to increase the sample size and to design a randomized controlled trial, as well as to compare Piezosurgery with traditional surgery, evaluating the degree of patient comfort both during surgery and in the post-operative period.

### Ethics approval

All procedures performed in studies involving human participants were in accordance with the ethical standards of the institutional and/or national research committee and with the 1964 Helsinki Declaration and its later amendments or comparable ethical standards. The study was approved by the regional Ethical Review Board of the Umberto I General Hospital (No. 3755).

### Consent to participate

Informed consent was obtained from all individual participants included in the study.

### Consent to publish

Patients signed informed consent regarding publishing their data and photographs.

## Data Availability

The datasets used and/or analyzed during the current study are available from the corresponding author on reasonable request.
