# Author Correction: Periodontal evaluation of palatally impacted maxillary canines treated by closed approach with ultrasonic surgery and orthodontic treatment: a retrospective pilot study

**DOI:** 10.1038/s41598-021-99454-y

**Published:** 2021-09-30

**Authors:** Camilla Grenga, Rosanna Guarnieri, Vittorio Grenga, Mauro Bovi, Serena Bertoldo, Gabriella Galluccio, Roberto Di Giorgio, Ersilia Barbato

**Affiliations:** 1 Rome, Italy; 2 Department of Oral and Maxillofacial Sciences, School of Dentistry, Sapienza, University of Rome, Rome, Italy

Correction to: *Scientific Reports* 10.1038/s41598-021-82510-y, published online 02 February 2021.

The original version of this Article contained an error in the Statistical analysis section, where

“Subsequently, for each tooth and for each site, a comparison was made between the quantitative distributions of the test group samples with control group samples through a Student’s t-test for independent samples with the hypothesis of homoscedasticity.”

now reads:

"Subsequently, for each tooth and for each site, a comparison was made between the quantitative distributions of the test group samples with control group samples through a paired Student t-test with the hypothesis of homoscedasticity."

The original Article has been corrected.

